# Comment on Sinkovič et al. Isotope Fingerprints of Common and Tartary Buckwheat Grains and Milling Fractions: A Preliminary Study. *Foods* 2022, *11*, 1414

**DOI:** 10.3390/foods11172626

**Published:** 2022-08-30

**Authors:** Micha Horacek, Andrew Cannavan

**Affiliations:** 1 Department of Lithospheric Research, Vienna University, 1090 Vienna, Austria; 2 HBLA & BA of Pomology and Oenology, 3400 Klosterneuburg, Austria; 3 Food Safety and Control Section, Joint FAO/IAEA Centre of Nuclear Techniques in Food and Agriculture, Department of Nuclear Sciences and Applications, International Atomic Energy Agency, Vienna International Centre, 1400 Vienna, Austria

We read with interest the publication by Sinkovic et al. [1] on the isotope patterns of Slovenian buckwheat. As research on buckwheat has up to date been neglected, this study certainly is an interesting first report of preliminary results from this commodity. However, we have noticed some errors and inconsistencies that we deem need correction.

The authors describe, for some of their samples, different sub-samples that they compare with the whole grain, i.e., hulls, semolina and light flour. Buckwheat hulls are the husks of the buckwheat grains (https://dict.leo.org, accessed on 18 August 2022, https://en.wikipedia.org/wiki/Buckwheat, accessed on 18 August 2022). Semolina is a coarse flour with a particle size between 0.3 to 1 mm (i.e., fine to coarse semolina). “Light flour” is comprised of milling-fraction particles below 0.15 mm (definition of flour). The definitions here are public knowledge and openly available, as unfortunately no exact definitions (for example: what exactly is “light flour”) are given in the article. From the description, it is unclear whether the hulls are removed from the whole grain before milling (and before processing for isotope analysis) or if the hulls were milled together with the grain. In this comment, the former is assumed, as the hulls are not consumed. Further uncertainty exists with respect to the bran, whether it was removed in the milling process, or remained as part of the milled fractions.

One concern is the data of organically grown buckwheat (of two cultivars) harvested over three consecutive years from the same field(s?). The authors analyzed the entire (whole) grain, buckwheat hulls and two milling-fractions (semolina and “light flour”). This means that the entire grain sample (whole grain) was analyzed together with two subsamples of each sample, i.e., the two grain-sizes of the (dehusked) grain (one coarse and one fine) consisting mainly of starch, and the hulls consisting mainly of fibers. Neither the fibers nor the starch contain nitrogen or sulfur, which means that these two elements are concentrated in the protein fraction of these (sub)samples, which should be slightly higher in the milled grain fractions than in the hull fraction, as in the grain embryo a large part of the grain protein is concentrated. In addition, a small amount of fat is present in the grain fraction (ca. 2%, mainly in the grain embryo, https://www.getreide.org/buchweizen.html, accessed on 18 August 2022, https://en.wikipedia.org/wiki/Endosperm, accessed on 18 August 2022). The authors state that the values of the different fractions (including hulls) and the whole grain do not show statistically significant differences for their respective C- and N-isotopes, only for the S-isotopes. Still, we find it very intriguing that the ^13^C- and ^15^N-ratio in the hulls consistently show the lowest values with respect to the whole grain and the two milling-fractions, even though these differences are not significant by the Kruskal–Wallis test. Also interesting are the d^13^C-values of the common buckwheat (sub)samples, which are consistently lower than the tartary buckwheat samples, even though they are slightly beyond the significance level at 0.05.

Sinkovic et al. [1] explain the ^13^C data as follows: “Generally, d^13^C values in plants reflect the signature of the soil organic C on the surface horizon of undisturbed (virgin) soils”. Unfortunately, they do not cite any reference, nor do they provide any evidence in support of this explanation, as this is (to our knowledge) a completely new hypothesis. Conventionally, ^13^C-values of plants are explained by the plant (C_3_-, C_4_-, CAM-photosynthesis) metabolism [2], taking up CO_2_ from the ambient atmosphere, and by water availability regulating the opening or closure of the plant leaf stomata [3] and references therein). Thus, unlike the explanation by Sinkovic et al. [1], “undisturbed (virgin) soils” do only influence the ^13^C-pattern of the plants growing on these soils by their water retention capacities. Instead, plants growing on (pristine) soils influence the ^13^C-pattern of the organic soil fraction by their produced plant litter accumulating on/in the surface soil. Consequently, the first growing of C_4_-plants on undisturbed soil previously dominated by C_3_-plants does not produce C_4_-plants with C_3_-plant ^13^C-isotope patterns, as Sinkovic et al. [1] suggest. Instead, these C_4_-plants growing on “undisturbed (virgin) soils” will also show typical C_4_-plants ^13^C-isotope patterns, solely influenced by water availability and occurrence of drought stress and the genetically determined sensitivity of these plants to drought stress (and also, if grown under controlled/closed conditions such as in a glass house, by the isotopic composition of the ambient atmospheric CO_2_).

As no further information is available concerning the isotope measurements, we would suggest that the most likely explanation for the ^15^N-pattern (showing the lowest values in the hull (sub)samples) is the lower concentration of N in the hulls leading to lower isotope values, assuming that for the analyses similar sample amounts were weighed-in for the four (sub)sample types, as stated by Sinkovic et al. [1]. The observed ^13^C-pattern of the sub-samples cannot be explained due to the isotope analysis, as all (sub)samples contain a high amount of C. In addition, the fat content of the grains does not have any relevance, as it would lead to lower ^13^C-values of the grain (sub)samples than the hulls. Instead, we speculate that hulls and grains might develop during different times of the buckwheat growth period with the hulls growing earlier than the grains, and thus changing environmental conditions resulting in no, or lower, drought stress during the earlier growth of the hulls and slightly higher drought stress resulting in slightly increased (but still low) ^13^C-values during formation of the grains [4,5]. However, other explanations are possible, including the complete and total coincidence or random occurrence of these ^13^C- and ^15^N-patterns. The lower ^13^C-values of the organic common buckwheat samples with respect to the tartary buckwheat seem to reflect some local differences in water availability, as no systematic difference between these two cultivars can be identified in the conventional samples.

The sulfur isotope results (Figure 1) are less clear, as one hull result was regarded as unreliable by the authors (due to the low S-concentration in this sample) and not considered for interpretation. Of the remaining three samples, one shows an elevated ^34^S-value for the hull sample with respect to the respective other (sub-) samples, whereas two samples have identical values for the hulls and the whole grain samples. Yet, for the sulfur isotope system, Sinkovic et al. [1] report a statistically significant variation in the hulls with respect to the other sample types. The authors explain the observed pattern by different pathways of S in the plants´ metabolism and identify/interpret the outer layer of buckwheat containing higher amounts of sulfur also as containing a higher ^34^S-ratio in comparison to the “… lower content of S enriched in lighter S isotopes […] located in the semolina fraction”. However, for us no solid evidence is provided for the interpretation of the S-isotope pattern Sinkovic et al. [1] report. As the outer layer of the buckwheat (enriched in S content) still belongs to the seed embryo (the cotyledons having the highest S-concentration [6] and is part of the endosperm) it is in no way related to the hulls. Furthermore, all sulfur enters the plant as bio-available sulfate, which later on, within the plant, is either stored as sulfate or quantitatively reduced and incorporated. However, the two ways of sulfur assimilation Sinkovic et al. [1] describe both belong to the reduced sulfate pathway [7]. Sinkovic et al. [1] incorrectly describe for both pathways that sulfate enters the embryo or seed coat/endosperm, whereas, in fact, for both pathways, the sulfur is reduced beforehand, and therefore no difference in sulfur assimilation (that potentially might result in S-isotope fractionation) should exist. Even, if these pathways should result in sulfur isotope fractionation, these differences would occur within the grain and not between grain and hulls. Thus, to us, these explanations do not make sense.

Furthermore, we cannot find any logical explanation for differences in ^34^S-values (Figure 1) between hulls and grain (sub-samples) or between semolina and the flour (as shown by Sinkovic et al. [1] for two of the investigated samples), as the latter sub-samples consist of exactly the same material, only in different milling-sizes. In our opinion, as described for the N-isotope values, problems with the S-isotope measurement of these samples due to low S-concentrations in the samples remain the most straightforward and likely explanation. All fractions contain low S-concentrations, with the hulls most likely possessing even lower concentrations than the other (sub)samples. Very low S concentrations might result in a deviating S-isotope ratio. Some evidence for this hypothesis is provided by the elevated ^34^S-value of the hulls of the one sample, which was excluded from further evaluation due to the small measurement peak and which just might be a more extreme result of low S concentrations in the (sub-) samples, and the fact that the S-isotope variations occur (besides the hulls (high values) and semolina (low values)) non-systematic. According to our interpretation, the semolina sub-samples might possess slightly higher S-concentrations than the other sample types, in that way explaining their consistent ^34^S-pattern.

The term “hull” normally refers to the husks, the tough outer layer of a grain outside the bran layer that is removed before milling. However, if “hull” is used in this paper to describe a milling fraction, it may refer to the skin of the grain. Parts of the skin of the grains are separated from the grain during the milling process, and together with (parts of) the grain embryo, they form the bran. In favor of this latter interpretation are: the description by Sinkovic et al. that can be understood to imply that the hulls are a milling fraction; the lower ^13^C-values of the hulls, which can then be explained by the fat from the grain embryo; and their interpretation of the S-isotope values, which might make slightly more sense. However, the latter still is not consistent, as in that case the two processes assumed by Sinkovic et al. [1] to produce different ^34^S-ratios (transporting S to the embryo and the grain skin) will be pooled together in the bran fraction. Arguing against this interpretation of “hull” (besides the incorrect use of this word) is the fact that bran contains ca. 15% protein and thus more sulfur than the other sample types, which obviously was not the case for the samples analyzed by Sinkovic et al. (as they even excluded one result because of low sulfur content).

The interpretation of very low δ^34^S-values (below ca. −20‰) in grain resulting from volcanic rocks, as mentioned by Sinkovic et al. [1], is highly unlikely, as volcanic rocks usually possess δ^34^S-values above this threshold value. The most probable sources are oxidized (biogenic) sulfides in sedimentary rocks (e.g., Hoefs [8], see Figure 2). Generally, knowledge about the bedrock geology and its (isotopic) characteristics is important when interpreting geogenic parameters [9]. 

A further problem of the article under consideration is the interpretation of differing isotope patterns of conventional and organic samples by the farming system. Even though the higher ^15^N-values might be an indicator for organic farming, these differences in ^15^N could potentially be due to the fact that these two different production types were produced at different localities and thus might be solely caused by differing environmental conditions at these two sites. The differing ^34^S-values of these two sites can, most likely, mainly be explained by environmental conditions (differing soil ^34^S-values). Even for a preliminary study, the evaluation of only two sites (and only one agricultural practice at each site) is not satisfactory and, in our opinion, does not provide enough comparable data to justify any interpretation.

In conclusion, the quite large variation in δ^34^S values within organic buckwheat samples (5.0–8.0 permil) is most likely explained by deviating (elevated) δ^34^S-values due to low S-concentrations in (sub-) and hull samples. The most reliable sample material seems to be the seminola samples, with reported values consistently between 5.0 and 5.5 permil (within the uncertainty of measurement and thus indicating homogenous conditions with respect to δ^34^S). All other sample types report larger ranges, with whole grain and light flour varying beyond 2 permil, whereas the hulls show a lower range (below 1.5 permil); however, with consistently elevated values (most probably due to representing the commodity with the lowest S-concentrations and thus influencing the ^34^S-analysis). We also propose that the lower N-isotope values of the hull samples could quite possibly be explained by lower N-concentrations than in the other sample types measured.

Inaccuracies: Sinkovic et al. [1] unfortunately report several incorrect numbers in their presented data (δ^34^S-values of the organic samples in their Table 2 [1] do not conform with the values in Table 1 in that publication, and thus the upper ranges of δ^34^S in Table 2 and also the lower range of δ^34^S in Tables 1 and 2 (tartary) and lower range of ^15^N (tartary) in Table 2 are not correct).

## Figures and Tables

**Figure 1:**
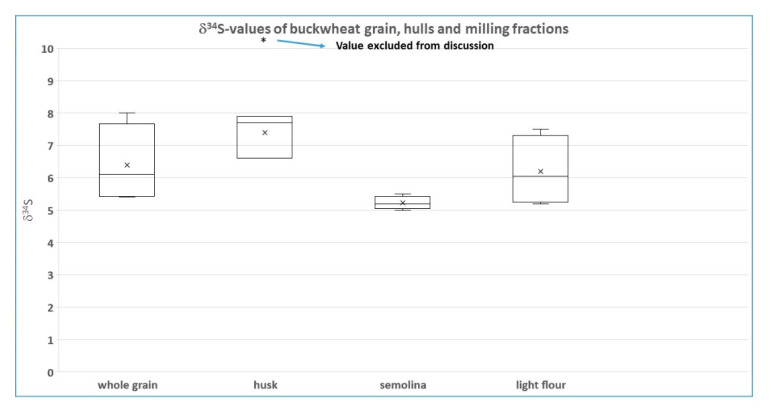
δ^34^S-values of the organic buckwheat (sub)samples collected over 2 years by Sinkovic et al. [1]. Asterisk marks the one (hulls)-sample discarded by the authors. Note the low variability of the semolina fraction.

**Figure 2:**
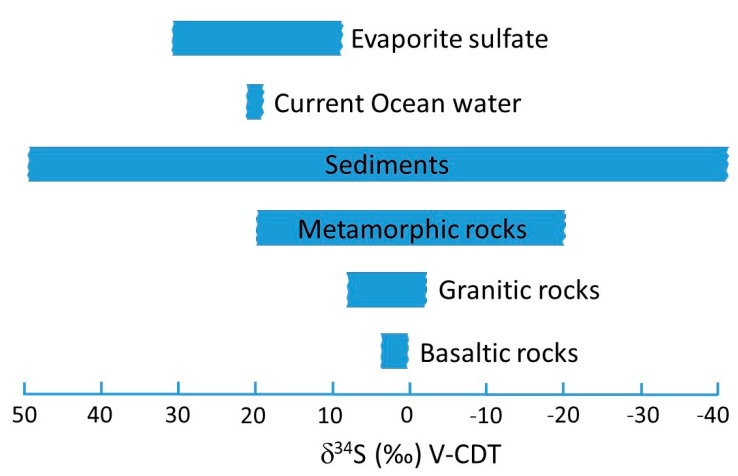
Sulfur isotope ranges in rocks, after Hoefs [8] and Horacek et al., 2010 [10], modified. Volcanic rocks belong to the groups “Basaltic rocks” and “Metamorphic rocks”, the latter group also representing acidic volcanic rocks from recycled continental crust. VCDT: Vienna Canyon Diablo Troilite.

## Data Availability

Data are contained within the article and present upon reasonable request from corresponding author.
